# Pharmacotherapy in COVID 19: Potential Impact of Targeting the Complement System

**DOI:** 10.3390/biomedicines9010011

**Published:** 2020-12-24

**Authors:** Courtney M. Barkoff, Shaker A. Mousa

**Affiliations:** The Pharmaceutical Research Institute, Albany College of Pharmacy and Health Sciences, 1 Discovery Drive, Rensselaer, NY 12144, USA; courtney.barkoff@acphs.edu

**Keywords:** COVID-19, SARS-CoV-2, complement, C3, C5, heparin, inflammation, coagulopathy, compstatin

## Abstract

Coronavirus disease 2019 (COVID-19), a respiratory illness caused by infection with severe acute respiratory syndrome coronavirus 2 (SARS-CoV-2), has claimed over one million lives worldwide since December 2019. The complement system, while a first-line immune defense against invading pathogens, has off-target effects that lead to increases in inflammation, tissue damage, and thrombosis; these are common, life-threatening complications seen in patients with COVID-19. This review explores the potential impact of complement activation in COVID-19 and possible treatments targeting the complement system.

## 1. Introduction

Coronavirus disease 2019 (COVID-19) is a respiratory illness caused by severe acute respiratory syndrome coronavirus 2 (SARS-CoV-2) infection [1] and was first recognized in December 2019 in Wuhan, China [2]. The virus quickly spread throughout the world and has been reported in over 180 countries [1]. As of December 14, 2020, there have been 73,188,395 cases and 1,627,783 deaths worldwide [3]. There is currently only one medication approved by the Food and Drug Administration (FDA) for the treatment of COVID-19 as well as two additional antiviral therapies available through emergency use authorization [1,2]. New data on treatment possibilities are emerging daily, and many investigations and clinical trials are underway. There is an urgent need for information on treatment of COVID-19. Many of the symptoms associated with COVID-19 can be related to immune response and complement activation. This review will summarize the impact of SAR-CoV-2 and emphasize the potential impact of complement activation in COVID-19 and possible treatments targeting the complement system. 

## 2. Search Strategy and Selection Criteria

References for this review were identified through searches of PubMed and Google Scholar. Search terms included “COVID,” “COVID-19,” “SARS-CoV-2,” “coronavirus,” “history,” “complement,” “treatment,” “C3,” “compstatin,” “C5,” “inhibition,” “heparin,” “anti-inflammatory,” “anticoagulation,” and “antiviral”. Articles resulting from these searches and relevant references cited in those articles were reviewed. Articles published in English, Chinese, and Italian were included. Articles published within the last 5 years were preferred, but older articles were not excluded if relevant. 

## 3. Coronaviruses

Coronaviruses are responsible for about 15% of common colds in adults and 35% of cases in respiratory infection epidemics [4]. Severe acute respiratory distress syndrome coronaviruses (SARS-CoV), H5N1 influenza A (avian flu), H1N1 2009 (swine flu), SARS-CoV-1 (SARS), and Middle East respiratory syndrome coronavirus (MERS-CoV) can cause acute lung injury and acute respiratory distress syndrome (ARDS), which may lead to pulmonary failure and possibly death [5]. SARS spread globally from China in 2003, causing pneumonia-like symptoms and ARDS, resulting in 8,000 cases and 775 deaths [5]. In 2012, MERS-CoV infected Saudi Arabian nationals causing pneumonia, ARDS, and renal failure, with 2428 cases and 838 deaths [5]. Both SARS and MERS-CoV have zoonotic origins tracing to the consumption of bats, and this is the suspected source of SARS-CoV-2 [5]. Coronaviruses are mainly spread from person to person through respiratory droplets or direct contact with infected persons, although transmission can also occur from contact with contaminated surfaces such as plastic, stainless steel, and cardboard [2,4]. 

Coronaviruses are RNA viruses belonging to the Coronaviridae family of the Nidovirales order [5]. Most coronaviruses share a similar molecular structure signified by glycoprotein spikes on their outer surfaces that attach to host cells for viral entry via virus-specific receptor-binding domain (RBD) [4,5]. The RBD of SARS-CoV-1 and SARS-CoV-2 has a high affinity for the angiotensin-converting enzyme-2 (ACE-2) receptor, especially in the lungs [4]. Upon viral binding to ACE-2 receptors in the lungs, immune and inflammatory cascades are triggered [4]. Although much is still unknown about SARS-CoV-2, it shares many genomic similarities with SARS-CoV-1, making their biochemical effects and pathogenesis also similar [4]. 

## 4. The Complement System

**Figure 1:**
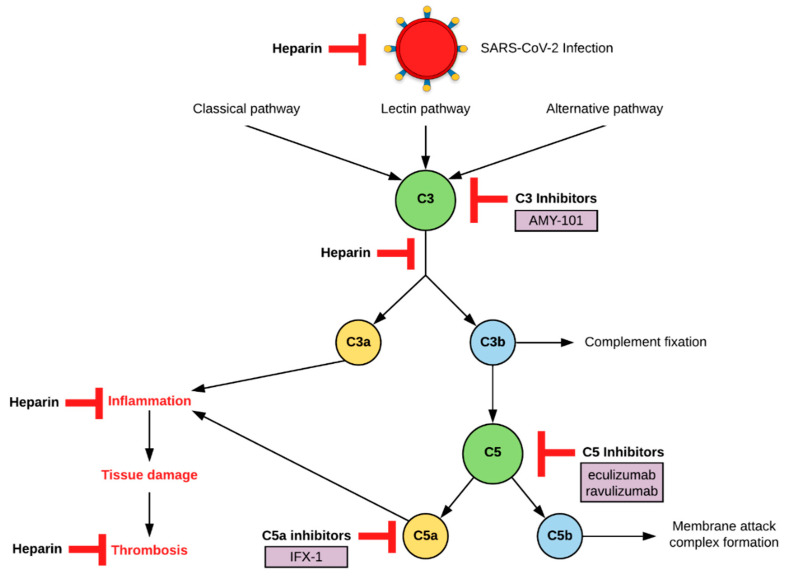
Complement activation pathway and location of action of anti-complement drug therapies. T-arrow indicates inhibition or interference of pathway at point of intersection.

The complement system (Figure 1) is one of the immune system’s first lines of defense against invading pathogens [6]. Complement consists of a group of soluble proteins that function to coat the surface of a pathogen to facilitate phagocytosis, and it is activated through a cascade of enzymatic reactions. 

There are three pathways from which the complement system can be activated: the alternative, lectin, and classical pathways [6]. All three pathways converge on the activation of the main complement component C3. Upon cleavage of the C3 protein by proteases known as C3 convertases, fragments C3a and C3b are created. C3a is responsible for recruiting effector cells to the site of infection and C3b covalently binds to the pathogen, tagging it for destruction. This tagging is known as complement fixation. C3b also goes on to cleave complement component C5 to fragments C5a and C5b. The C5b fragment initiates the formation of the membrane attack complex (MAC), which forms holes in the membranes of pathogens. While C3b and C5b contribute to the immune recognition and destruction of pathogens, their counter-components C3a and C5a may contribute to host damage. Both C3a and C5a cause increased inflammation at the site of activation. C5a and the MAC upregulate neutrophil activation and inflammation, which leads to endothelial cell damage [7]. Damage to the cells of blood vessels activates the coagulation system and promotes blood clotting [6]. 

The primary site of infection and complement activation with SARS-CoV-2 is the lungs, and immune damage occurs to airway epithelial cells and vascular endothelial cells, leading to ARDS and thrombotic complications [8]. SARS-CoV-2 spike protein has been shown to directly activate the alternative complement pathway [9]. SARS-CoV, which is closely related to SARS-CoV-2, has been found to be exacerbated by the activation of C3 [8]. SARS-CoV-infected mice deficient in C3 exhibited less respiratory dysfunction despite equivalent viral loads within the lungs [10]. A pre-print study has shown that lung biopsies from patients with severe COVID-19 reveal evidence of widespread complement hyper-activation, but this information has not yet been peer reviewed [11]. 

Given what is known about the SARS-CoV-2 and the body’s immune response to it, the complement system is a possible mechanism to combat COVID-19. The damage done by the complement system’s reaction to SARS-CoV-2 may be mitigated through inhibition of C3, C5, or their fragments. Blocking C3 would also block the downstream production of C3a and C5a [8]. Blocking C5 may provide partial inhibition and allow residual terminal pathway activity to skew efficacy [8]. Both the genetic absence of C3 and blockade of downstream C5 components have shown therapeutic benefit through prevention of monocyte and neutrophil activation and immune cell infiltration in the lungs, decreasing the damage from immune-mediated inflammation [12]. 

## 5. COVID-19 Clinical Presentation and Progression 

Upon exposure to SARS-CoV-2, there is a two to 14 day asymptomatic incubation period during which the virus may be spread [13]. Although the majority of patients infected with SARS-CoV-2 exhibit mild to moderate symptoms, up to 15% of patients progress to severe disease and require oxygen support, and up to 5% of patients become critically ill with possible respiratory failure, ARDS, sepsis, thromboembolism, and multi-organ failure [2]. Similar to SARS and MERS, the most common symptoms of COVID-19 are fever, fatigue, cough, and shortness of breath [13,14]. Other commonly reported symptoms of COVID-19 include diarrhea, headache, and loss of taste and smell [1]. Common laboratory findings in patients with COVID-19 include leukopenia and lymphopenia, as well as elevated markers of inflammation such as C-reactive protein, D-dimer, and ferritin [1]. Chest x-ray often reveals bilateral multi-focal opacities in the lung and CT of the chest typically shows bilateral peripheral ground-glass opacities and the development of areas of consolidation as the disease progresses [1]. 

Those at risk for severe and possibly fatal disease include those over the age of 65, those living in nursing homes or long-term care facilities, or people of any age with certain comorbidities. At-risk conditions include hypertension, cardiovascular disease, diabetes, chronic respiratory disease, cancer, renal disease, and obesity [1]. Among patients who develop severe disease, there is a possibility for rapid deterioration approximately one week after onset of symptoms [14]. On average, patients who develop severe disease develop dyspnea within five to eight days, ARDS within eight to 12 days, and require ICU admission within 10–12 days [14]. Of patients requiring hospitalization, approximately 30% require admission to the ICU [14]. Mortality rates among ICU patients with severe COVID-19 have been variable, ranging from 40–80% [14]. 

## 6. Current Treatments under Investigation

Although the only drug approved by the FDA to treat COVID-19 is remdesivir, there are many treatment investigations and clinical trials still taking place. Possible treatments are broken down into antiviral and immune-based therapies [1]. Relevant treatment investigations, rationale, and clinical results can be found in Table 1.

## 7. Complement as a Therapeutic Target

### 7.1. C3 Inhibition

Compstatin is a highly selective inhibitor of C3 [52]. A potent compstatin-based C3 inhibitor, AMY-101, has been developed [12]. The molecule is a small peptide that is selective for human and primate C3. AMY-101 is currently in phase II clinical trials after showing favorable safety and tolerability in humans in phase I trials. A case report has demonstrated that AMY-101 may facilitate clinical improvement in severe COVID-19 patients [12]. The case presents a 71-year-old male patient diagnosed with ARDS caused by SARS-CoV-2. The patient was eligible as a candidate for experimental treatments of COVID-19, including compassionate use of AMY-101. The therapy was administered as a one-time loading dose, followed by 13 daily maintenance doses for a total of 14 days of treatment. No side effects were recorded after the loading dose and no infusion reactions were reported during the entire duration of therapy. The patient showed dramatic clinical improvement within 48 h of AMY-101 treatment, demonstrated by significantly improved respiratory performance and a gradual decrease in oxygen requirements. By day 19 after initiation of AMY-101 treatment initiation, the patient was afebrile with no respiratory symptoms and was without need for oxygen support. This case report preliminarily demonstrates the safe and effective use of AMY-101 in the treatment of severe COVID-19 and reinforces the need for further clinical studies of C3 inhibition as anti-inflammatory therapy.

### 7.2. C5 Inhibition

C5 inhibition has been used clinically for 15 years in the setting of paroxysmal nocturnal hemoglobinuria (PNH), a rare blood disorder characterized by complement-dependent hemolysis [53,54]. The C5 inhibitors eculizumab (Soliris) and ravulizumab-cwvz (Ultomiris) have both been proposed in the treatment of ARDS in COVID-19 [55,56]. Eculizumab, a long-acting humanized monoclonal antibody against complement C5, is currently available for compassionate use in COVID-19 under an expanded access program [55]. Preliminary results of patients with severe COVID-19 treated with eculizumab yielded positive results. In a case report of four patients treated with eculizumab off-label, all patients successfully recovered after treatment with a noted drop in inflammatory markers [57]. The Eculizumab (Soliris) in COVID-19 Infected Patients (SOLID C-19) trial is an ongoing compassionate use study [58]. A preliminary case report demonstrates the safe and effective use of eculizumab in a 44-year-old female patient with severe COVID-19 pneumonia on invasive mechanical ventilation [58]. Upon treatment with eculizumab, the patient’s chest x-ray and clinical condition improved. The patient continued to improve with consecutive doses and was able to be extubated 14 days later after four doses of eculizumab. These preliminary results are promising until more data become available. Ravulizumab-cwvz is a long-acting C5 complement inhibitor with an upcoming phase III clinical trial for COVID-19 severe pneumonia [56,59]. Based on anecdotal information from compassionate use cases, the phase III controlled clinical trial will assess 28-day survival among patients with severe COVID-19 receiving ravulizumab with current best supportive care or best supportive care alone. The study is expected to have primary outcome data by May 2021 and to be completed by August 2021 [56].

Results of a recent exploratory study comparing the efficacy of eculizumab to AMY-101 in independent cohorts reported clinical improvement in both groups of patients [60]. Three patients with severe COVID-19 ARDS were given AMY-101 via continuous IV infusion, while 10 patients with COVID-19 enrolled in a phase I/II single-arm clinical trial were given eculizumab IV once weekly. All treated patients required oxygen support prior to treatment initiation. Both treatments resulted in sustained anti-inflammatory response marked by reductions in C-reactive protein and IL-6, improvement in lung function, and resolution of ARDS. C3 inhibition with AMY-101 resulted in further reduced neutrophil counts, serum LDH levels, and lymphocyte recovery. These early results demonstrate not only the role of complement in COVID-19, but the utility of C3 and C5 as therapeutic targets to improve patient outcomes.

Clinical trials have begun for monoclonal anti-human C5a antibody, IFX-1, in patients with COVID-19 with severe pneumonia [61]. IFX-1 is not thought to impact formation of the MAC. IFX-1 does not currently have any approved indications but is in development to treat inflammatory conditions. According to preclinical data, IFX-1 controls inflammatory-related tissue and organ damage [61]. Phase II/III trials investigating the use of IFX-1 in COVID-19 are currently underway with results expected in mid-2021 [62]. Preliminary results are reported to be positive, with IFX-1 treatment associated with a trend in lower 28-day all-cause mortality compared to best supportive care alone [63]. Treatment with IFX-1 has also shown trends of maintained kidney function, faster lymphocyte count normalization, and greater reduction in LDH in patients with severe COVID-19 [63].

Avdoralimab is a fully human monoclonal antibody that blocks the C5a receptor [64]. By preventing the binding of C5a to the receptor, avdoralimab has the potential to reduce the C5a mediated inflammatory response to SARS-CoV-2 in the lungs [64]. Double-blind, randomized phase II trials, named FOR COVID-19 Elimination (FORCE), began in April 2020 [65]. The trial compares avdoralimab against placebo in two cohorts of patients with COVID-19: those requiring supplemental oxygen and those requiring mechanical ventilation. Results of the FORCE trial are expected at the end of 2020.

### 7.3. Heparin

Heparin is a familiar anticoagulant with clinical use dating back to the 1920s [66]. It achieves anticoagulation through antithrombin dependent inactivation of thrombin and activated coagulation factor Xa [67]. It also prevents fibrin formation and inhibits thrombin-induced activation of platelets and other coagulation factors [67].

**Figure 2:**
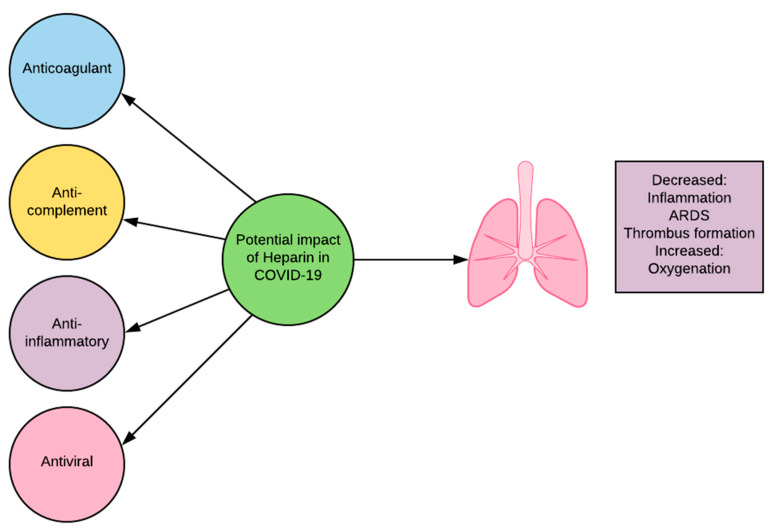
Potential impact of heparin in COVID-19.

The utility of heparin in COVID-19 is not limited to its anticoagulant activity (Figure 2). Heparin has been shown to also have anti-complement, anti-inflammatory, and anti-viral properties. Heparin interferes with C3 convertases and with the function of late components of the complement system [68]. It can do this independently of antithrombin binding activity [68]. The inhibition of C3 convertase blocks the cleavage of C3, preventing the creation of detrimental fragments C3a and downstream C5a. Heparin inhibits inflammation through alteration of pro-inflammatory protein activity and prevention of adhesion and influx of inflammatory cells in affected areas [69]. Heparin interacts directly with viral particles and has been shown to bind to the SARS-CoV-2 S1 Spike RBD, causing significant structural change and alteration of function [70]. Coronavirus surface proteins are known to interact with heparan sulfate, a glycosaminoglycans (GAG) closely related to heparin, in order to attach to target cells [70,71]. Heparin may act as a competitive inhibitor for these sites, decreasing viral attachment to host cells [72]. The ability of heparin to decrease complement activation, inflammation, and viral infiltration makes it a promising candidate for clinical review in COVID-19.

Heparin use in clinical trials has been divided into prophylactic or therapeutic dosing in the treatment of COVID-19 associated thrombosis. A retrospective study by Tang et al. reviewed data from 449 patients with severe COVID-19 [73]. All patients received antiviral therapy with supportive care and 99 patients also received low molecular weight heparin (LMWH) at prophylactic doses. Although no difference was found in 28-day mortality overall between the groups, heparin receivers did have lower 28-day mortality if they had a sepsis-induced coagulopathy (SIC) score ≥4 or D-dimer greater than six times the upper limit of normal. It was concluded that LMWH at prophylactic doses may be associated with improved prognosis in patients with severe COVID-19 meeting SIC criteria or with markedly elevated D-dimer.

There is limited data available for the outcomes of patients with COVID-19 receiving therapeutic doses of heparin. One cohort study of 2,773 patients found that of patients on invasive mechanical ventilation (*n* = 395), anticoagulation was associated with an in-hospital mortality of 29.1% [74]. Patients who did not receive anticoagulation (*n* = 1987) had an in-hospital mortality of 62.7%. The study also found that patients were more likely to require invasive mechanical ventilation if they received anticoagulation [74]. A retrospective, observational study of 2,075 patients with COVID-19 compared mortality between patients who received heparin (*n* = 1734) versus those who did not (*n* = 341) [75]. The data were adjusted for age, gender, signs of increasing severity (temperature greater than 30° C or oxygen saturation less than 90%), and other COVID-19 treatments. Heparin was associated with lower mortality when the model was adjusted for age and gender and remained significant when additionally adjusted for severity and then other treatments. These promising observational results highlight the need for further, controlled trials of heparin use in COVID-19.

Although promising, the use of heparin in COVID-19 is not without safety concerns. Therapeutic dosing of heparin is associated with a 10–15% risk of significant bleeding [69]. Another concern is the rare risk for heparin-induced thrombocytopenia. Although excess coagulation can cause many complications, coagulation in the alveoli and airways is protective to isolate pulmonary pathogens and prevent infection. Heparin also has effects on various growth factors that may result in protection or harm to organs [69]. More information is needed before a recommendation may be made one way or the other on the use of heparin in patients with COVID-19.

## 8. Conclusions

While there are many promising treatment options being investigated, the need for further discovery and understanding of drugs to treat COVID-19 is paramount. Until the infection can be effectively prevented, proper diagnosis and drug therapy remain on the forefront of treatment. Diagnostic testing and clinical presentation should be used to guide treatment so patients are receiving the best therapy, offering them the best chance at survival and recovery. The complement system is key in the body’s immune response to invading pathogens, its unchecked activation may lead to serious host damage and organ dysfunction. Targeting complement has been demonstrated to be effective in the treatment of other inflammatory conditions and may be the key to relieving immune-mediated lung inflammation and damage in COVID-19. Preliminary data suggest the successful use of several anti-complement drugs in patients with severe COVID-19 pneumonia. Regulating complement activation in response to SARS-CoV-2 has the potential to alleviate respiratory symptoms and prevent disease progression. This has the power to improve patient outcomes and save lives.

Beyond COVID-19, complement is a possible target for many immune-mediated inflammatory conditions. Complement plays a contributory role in many auto-immune diseases, such as systemic lupus erythematosus, rheumatoid arthritis, and antiphospholipid antibody syndrome [76]. Further demonstrated use of anti-complement therapies in COVID-19 may open the door for their investigation in additional settings.

## Figures and Tables

**Table 1:** Current SARS-CoV-2 and COVID-19 treatment investigations, rationale, and clinical data.

Drug	FDA Approved Indication	Proposed COVID-19 MOA	ADEs	Possible DDIs	Clinical Data/ Limitations
**Antiviral Therapies**					
Chloroquine	Malaria	● Prevents virus/cell binding and fusion [15,16,17] ● Immunomodulatory effects decrease inflammatory cytokines [16]	● QTc prolongation ● GI effects	● Other QTc prolonging drugs ● CYP2D6 inhibitor ● P-gp inhibitor	● No benefit found: ○ High dose vs. low dose CQ [18] ○ CQ vs. LPV [19] ○ HCQ vs. HCQ + AZM vs. no HCQ [20] ○ HCQ vs. SOC [21] ○ HCQ vs. no HCQ [22] ● Problems with study design (e.g., small sample sizes, extremes in patient demographics, differences in standard of care definitions) [1]
Hydroxy-chloroquine	Malaria, Lupus, RA				
Remdesivir (RDV)	COVID-19	● Adenosine nucleotide analog prodrug [23] ● Inhibits viral-dependent RNA polymerase [23]	● Elevated LFTs ● Renal toxicity associated with drug vehicle ● GI symptoms	● Strong induction of P-gp will reduce levels	● First to show clinical benefit of a pharmacological treatment for COVID 19 [1] ○ RDV vs. placebo [24] ○ Patients recovered 4 days faster on average [24] ○ Mortality rate 8% (RDV) vs. 11·5% (placebo) [24] ● RDV compassionate use [25] ○ Less oxygen support needed, no control [25] ● Some small sample sizes, uncontrolled cases [1]
Favipiravir (FPV)	None [26]	● Purine nucleoside analogue prodrug [27] ● Competitive inhibitor of RNA-dependent RNA polymerase [27]	● Diarrhea [28] ● Liver injury [28] ● Poor diet [28]	● Avoid administration with aldehyde oxidase inhibitors [26] ● CYP2D8 inhibitor [26]	● FPV vs. LPV [27] ○ Reduction in median time to viral clearance ○ 4 days (FPV) vs. 11 days (LPV) ○ Fewer adverse events than LPV
Protease Inhibitors (Lopinavir, Ritonavir)	HIV	● Possible SARS-CoV-2 protease 3CLpro inhibition [29]	● GI effects ● Elevated LFTs ● QTc prolongation	● Lopinavir: CYP3A4 inhibitor and substrate ● Ritonavir: CYP3A4 and 2D6 inhibitor and substrate, UGT1A1 inducer, inducer of CYP1A2, 2C8, 2C9, 2C19	● No virologic or clinical benefit found ○ LPV vs. SOC [1] ● Limitations (e.g.,. small sample sizes, unblinded, underpowered) [1]
Ivermectin	Parasitic infections	● Inhibits host nuclear transport proteins, preventing viral mediated antiviral suppression	● Rash ● Dizziness	● Few	● Retrospective analysis ● Non-standardized timing of interventions
**Immune-Based Therapies**					
Convalescent Plasma	None	● Contains antibodies against SARS-CoV-2 [30]	● Treatment-associated acute lung injury and circulatory overload [31] ● Hypersensitivity reactions ● Transfusion risks	● Dedicated IV line	● Limited data [1] ○ Small sample sizes ○ Retrospective cohort studies ○ Case series ○ Case reports
Immune Globulins: SARS-CoV-2 specific	None	● Concentrated antibodies against SARS-CoV-2 and/or other pathogens [1]	● Thrombotic events ● Renal dysfunction ● Infusion reactions	● May interfere with response to other vaccines	● SARS-CoV-2 specific: no clinical data [1] ● Non-SARS-CoV-2 specific: Limitations in study design (e.g.,. non-randomized, older patient age, higher population of patients with severe disease) [32]
Non-SARS-CoV-2 specific	Immune disorders, prophylaxis of bacterial and viral disorders				
Mesenchymal Stem Cells	None	● Hypothesized to reduce acute lung injury and inhibit cell mediated inflammatory response [1]	● Unpredictability of stem cell activity ● Tumor growth ● Infection ● Thrombus formation ● Site reactions	● Dedicated IV line	● Small, non-randomized studies [1] ● No statistical significance [1]
Interferons: Alpha	Leukemia, melanoma, lymphoma, HBV, HCV	● Antiviral ● Antiproliferative ● Immuno-modulatory [33,34,35,36]	● Flu-like symptoms ● Injection site reactions ● Altered LFTs ● Worsening depression [35,37]	● Low potential ● CYP1A2 inhibition with IFN-a	● No clinical data for COVID-19 [1] ● Mixed/limited data in MERS [38,39,40,41] ● Inhaled IFN-b in other conditions [42,43]
Beta	Multiple sclerosis				
JAK Inhibitors (Baricitinib)	RA [44]	● Inhibition of kinases that regulate endocytosis [1] ● Predicted to interfere with endocytosis in alveolar epithelial cells [45]	● Lymphoma ● Thrombosis ● GI perforation ● LFT changes ● Herpes simplex ● Herpes zoster	● Modify dose with strong OAT3 inhibitors	● No data for COVID 19, SARS, or MERS [1]
IL-1 inhibitors (Anakinra)	RA	● Competitive binding to IL-1 receptor	● Neutropenia ● Anaphylaxis ● Injection site reactions Infusion reactions	● Avoid with TNF inhibitors due to risk of infection	● No data for COVID 19, SARS, or MERS [1]
IL-6 inhibitors (Sarilumab, Siltuximab, Tocilizumab)	Sar: RA [46] Sil: Multicentric Castleman Disease Toc: Cytokine release syndrome, RA	● Monoclonal Antibodies [1] I● L-6 receptor antagonists [47,48]	Neutropenia GI perforation Infusion reactions LFT changes	● Elevated IL-6 may downregulate CYP	● Sar, Toc: Underway [49] ● Sil: single center observational study; mixed results [1] ● Toc: uncontrolled retrospective cohort; small sample size; no control; positive results [50]
**Other**					
Dexamethasone [51]	Asthma, inflammatory disorders	● Synthetic adrenal corticosteroid ● Anti-inflammatory effects	● Hypertension ● Hyperglycemia	● CYP3A4 inducer	● RECOVERY Trial ○ Reduced death by 1/3 in patients on respirator, by 1/5 in patients on ventilator [51] ○ Positive effects in critically ill patients, not in mild cases [51] ● Delayed viral clearance in SARS and MERS [1]

Abbreviations: FDA: Food and Drug Administration, MOA: mechanism of action, ADE: adverse drug event, DDI: drug-drug interaction, RA: rheumatoid arthritis, CQ: chloroquine, GI: gastrointestinal, LPV: lopinavir, HCQ: hydroxychloroquine, AZM: azithromycin, SOC: standard of care, LFT: liver function tests, RDV: remdesivir, FPV: favipiravir, HBV: hepatitis B virus, HCV: hepatitis C virus, IFN: interferon, JAK: janus kinase, IL: interleukin.
